# Whole-Genome Sequence-Based Diversity of *Mycobacterium tuberculosis* Strains Isolated from a Central Western Region of Mexico

**DOI:** 10.3390/pathogens14060548

**Published:** 2025-05-31

**Authors:** Andrea Monserrat Negrete-Paz, Gerardo Vázquez-Marrufo, Adrián Rodríguez-Carlos, Bruno Rivas-Santiago, Ma. Soledad Vázquez-Garcidueñas

**Affiliations:** 1 División de Estudios de Posgrado, Facultad de Ciencias Médicas y Biológicas “Dr. Ignacio Chávez”, Universidad Michoacana de San Nicolás de Hidalgo, Morelia C.P. 58020, Michoacán, Mexico; andrea.negrete@umich.mx; 2 Centro Multidisciplinario de Estudios en Biotecnología, Facultad de Medicina Veterinaria y Zootecnia, Universidad Michoacana de San Nicolás de Hidalgo, Tarímbaro C.P. 58893, Michoacán, Mexico; gerardo.marrufo@umich.mx; 3 Unidad de Investigación Biomédica de Zacatecas, Instituto Mexicano del Seguro Social-IMSS, Zacatecas C.P. 98000, Zacatecas, Mexico; adrian.rodriguezca@imss.gob.mx (A.R.-C.); bruno.rivas@imss.gob.mx (B.R.-S.)

**Keywords:** tuberculosis, typing, drug resistance, whole-genome sequencing

## Abstract

Tuberculosis remains a significant health issue in Mexico, which has one of the highest incidence rates in the Americas. This study aimed to analyze the circulating sublineages, spoligotypes, drug resistance, and transmission patterns of *Mycobacterium tuberculosis* in Mexico’s Central Western region using whole-genome sequencing. Seventy-seven *Mycobacterium tuberculosis* strains underwent phenotypic drug susceptibility testing via MGIT. Genotypic resistance was assessed with TB-Profiler and Mykrobe, while phylogenetic relationships were reconstructed using Snippy and RaxML. SpoTyping identified circulating SITs and families, with a 5-SNP threshold defining genomic transmission clusters. The predominant sublineages were 4.1.1.3 (X-type, *n* = 19) and 4.1.2.1 (LAM, *n* = 11), with rare sublineages (EAI5, EAI2-Manila, and Beijing) also observed. Resistance to at least one first-line drug was found in 63.3% of strains, with streptomycin mono-resistance (24.5%) being notable. Multidrug-resistant TB was identified in 16.3% (*n* = 8) of strains. Five genomic clusters, involving 18.7% of strains, were identified. This study highlights the sublineage diversity in Mexico, emphasizing its importance in global databases and resistance research. The findings, such as SIT47 in GC1, underscore the value of localized genomic studies for effective TB control.

## 1. Introduction

Tuberculosis (TB), one of the oldest known human diseases, remains the deadliest infectious disease worldwide, even with the global use of a live attenuated vaccine and multiple antibiotics [1]. TB is caused by the bacteria *Mycobacterium tuberculosis* (MTB), a pathogen that spreads when infected people expel bacteria into the air. It is estimated that about a quarter of the global population has been infected with TB [2]. The disease typically affects the lungs (pulmonary TB) but can affect other sites as well (extrapulmonary TB). The World Health Organization (WHO) 2024 report on TB describes that a total of 1.25 million people died from this disease in 2023 (including 161,000 people with HIV). Globally, TB has likely regained its position as the leading cause of death from a single infectious agent, after being surpassed by coronavirus disease (COVID-19) for three years [2]. It was also a major cause of deaths related to antimicrobial resistance. In 2023, an estimated 10.8 million people fell ill with TB. In response, the WHO has been emphasizing improving TB detection, enhancing drug resistance management, and expanding vaccination coverage [2]. Although in the Americas, the TB incidence rate is relatively low in relation to other regions, it remains a public health concern. For the third year in a row, the Americas experienced a rise in TB incidence rates, partially due to COVID-19 pandemic-related disruptions in TB diagnosis and treatment that have not fully recovered. Additionally, TB mortality in the Americas showed a concerning trend, as deaths associated with this disease continued to rise in 2022 [3].

Countries in the Americas with the highest burden include Brazil, Peru, and Mexico. In Brazil and Mexico, TB prevalence is high in urban and underserved communities, where access to healthcare and diagnostic facilities is limited [3]. In 2023, Mexico reported an incidence rate of approximately 27 cases of TB per 100,000 people, amounting to around 34,000 new and relapse cases for the year [4]. Risk groups in Mexico include indigenous populations, inmates, and HIV/AIDS patients, who have higher incidence rates than the general population. Indigenous communities, for example, often experience TB at rates far above the national average due to factors like limited healthcare access and historical disparities in health services, as documented worldwide [5,6]. The Central Western Region of Mexico is densely populated by indigenous communities, characterized for the most part by a lack of adequate epidemiological surveillance for infectious diseases such as TB. Michoacán and Zacatecas states belong to this region with distinct epidemiological contexts, making them particularly relevant for TB research. Michoacán has significant Purépecha and Mazahua indigenous populations and serves as a major corridor for internal and international migration, potentially influencing TB transmission dynamics [7]. Additionally, there is no detailed epidemiological profile for TB in Michoacán, making surveillance particularly challenging. Zacatecas, while having established TB control programs, faces challenges related to high rates of migration to and from the United States. This migratory pattern is especially significant because studies in other regions of Mexico indicate that the MTB population is primarily composed of Latin American and Mediterranean (LAM) strains [8,9,10], which are part of the Euro-American lineage most widespread in the Americas. Research has shown that these LAM strains are predominant in Mexico and are associated with increased transmissibility and certain socio-environmental factors specific to the region [11]. The complex interplay between these LAM strains and the unique demographic and geographic characteristics of Michoacán and Zacatecas makes these states important but understudied regions for TB genomic surveillance, particularly as migration patterns may facilitate the introduction and spread of diverse MTB strains. Several molecular genetics methods have been employed in typing MTB strains, helping to categorize them based on genetic markers and variations, which is crucial for understanding their epidemiological patterns, resistance profiles, and transmission dynamics. The main typing methods are spoligotyping, the Mycobacterial Interspersed Repetitive Unit-Variable Number Tandem Repeat (MIRU-VNTR) pattern, and single nucleotide polymorphisms (SNPs) detection [12]. Spoligotyping is a widely used method to determine the family of MTB by analyzing the direct repeat (DR) region of the genome. It identifies the presence or absence of specific spacers and generates a unique spoligotype pattern used to assign strains to a family [13]. The MIRU-VNTR typing is based on variations in the number of repeats at certain loci in the genome. Different genotypes tend to have characteristic MIRU-VNTR patterns, which can help to identify the strain’s lineage. Whole-genome sequencing (WGS) provides the highest resolution by analyzing the entire genome of a strain [14]. This method identifies SNPs and other genetic variations that are specific to each lineage or sublineage. WGS enables accurate identification of all known MTB lineages, including detailed analysis of sublineages and mutations. Tools like TB-Profiler or Mykrobe [15,16] can use WGS data to classify strains into lineages, including the Euro-American (LAM), Beijing, and other lineages. It is especially useful for detecting drug-resistant strains and is highly effective for epidemiological studies, particularly for tracking the spread of TB and identifying the genetic markers of resistance. In Mexico, the MTB population structure has been mostly assessed by spoligotyping and MIRU-VNTR, but less is known regarding the genomic diversity associated with the country, and reports using higher resolution typing methods are needed to better understand the epidemiological pattern. To address this gap, we present the results of a genomic epidemiological study conducted in the Central Western region of Mexico, aiming to identify the circulating genotypes, analyze existing and active transmission clusters within the community, and assess the distribution of drug resistance while exploring SNP-based lineages.

## 2. Materials and Methods

### 2.1. Sample Collection

A total of 67 MTB strains obtained from Laboratorio Estatal de Salud Pública of Michoacán (*n* = 29) and the Biomedical Research Unit Zacatecas-IMSS (*n* = 38) were included in this study. The samples were retrieved from patients with bacteriological confirmation of TB. Additionally, 10 previously sequenced MTB strains (Bioproject: PRJNA880281) [17] isolated from the state of Michoacán were included in the analysis, resulting in a total of 77 samples. These pre-sequenced strains were isolated from the same geographical region (Michoacán state) and time period as the newly sequenced Michoacán strains, ensuring population consistency. The inclusion criteria and collection methods for all strains were identical, minimizing potential selection bias.

The study was conducted in accordance with the Declaration of Helsinki, and the protocol was approved by the National Scientific Research Committee-IMSS (R-2022-785-049) on 19 October 2022. Obtaining clinical samples and isolating *M. tuberculosis* strains from patients with suspected or confirmed tuberculosis is part of the Epidemiological Surveillance Program in Mexico; so, in this work, the management of each clinical record, the taking of samples, and the isolation of the analyzed strains were carried out in accordance with the Mexican Official Standard, NOM-006-SSA2-2013, for the prevention and control of tuberculosis.

### 2.2. Phenotypic Susceptibility

Antimicrobial susceptibility profiling was executed utilizing the fluorometric BACTEC MGIT 960 system (Becton-Dickinson, Franklin Lakes, NJ, USA). The analytical parameters adhered to internationally standardized critical concentration thresholds for first-line antituberculosis agents: isoniazid (INH) at a concentration of 0.1 μg/mL, rifampin (RIF) at 1.0 μg/mL, ethambutol (EMB) at 5.0 μg/mL, and streptomycin (STR) at 1.0 μg/mL. Pyrazinamide (PZA) susceptibility determination was conducted employing the BACTEC MGIT 960 PZA kit (Becton Dickinson, Franklin Lakes, NJ, USA) in accordance with the manufacturer’s specified protocol.

### 2.3. DNA Extraction

DNA was extracted from mycobacterial cultures grown on Lowenstein–Jensen medium using the phenol–chloroform method [18]. The concentration and quality of the DNA were measured using Nanodrop (Thermo Scientific, Waltham, MA, USA. DNA samples were shipped to SeqCenter, Pittsburgh, PA, USA, for whole-genome sequencing.

### 2.4. Whole-Genome Sequencing (WGS)

The genomic DNA underwent library preparation utilizing the dual-technology Illumina DNA Prep system (Illumina Inc., San Diego, CA, USA), which combines enzymatic tagmentation with PCR amplification. Custom-designed IDT unique dual indices of 10 base pairs facilitated sample identification. The protocol established a predetermined target fragment length of 280 base pairs without employing additional fragmentation techniques or implementing fragment size selection methodologies. Sequence acquisition was conducted on the Illumina NovaSeq X Plus platform (Illumina Inc., San Diego, CA, USA) configured for simultaneous processing of multiple indexed samples within shared flow-cell environments. This configuration generated bidirectional sequence information in the form of 151 base pair paired-end reads. Post-sequencing data processing, including sample demultiplexing, quality parameter assessment, and adapter sequence elimination, was accomplished using the bcl-convert1 software package (version 4.2.4, llumina Inc., San Diego, CA, USA) [19].

### 2.5. Bioinformatics Analysis

**Figure 1:**
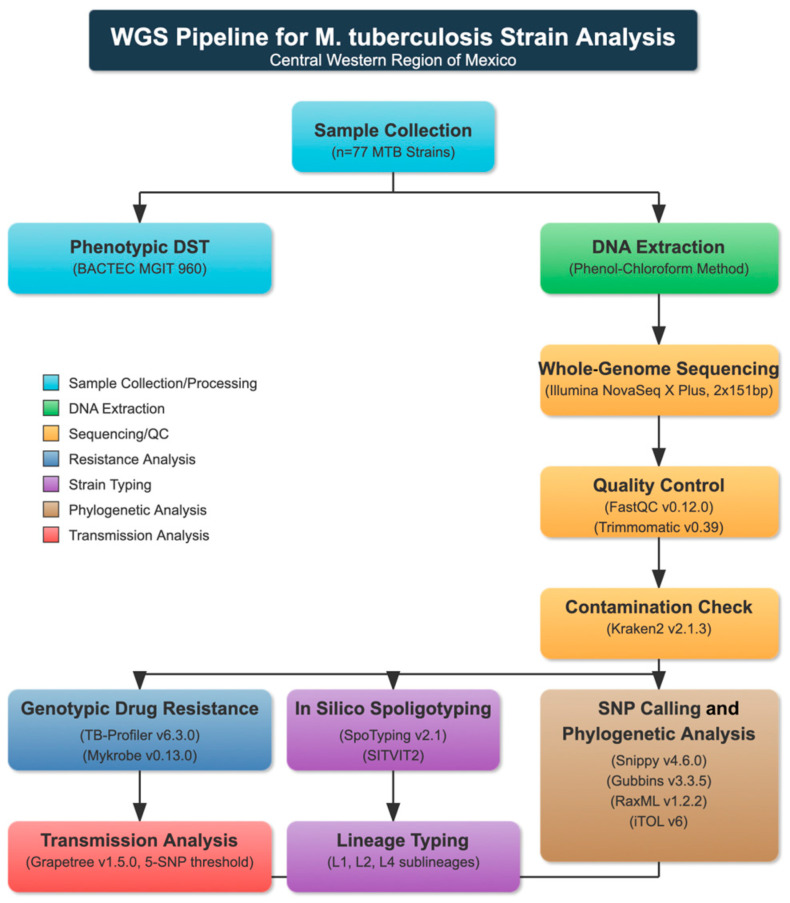
Pipeline for *M. tuberculosis* strain analysis from Central Western Region of Mexico.

Quality control and trimming to remove adapter sequences and low-quality reads were performed using FastQC (v0.12.0, Babraham Bioinformatics, Cambridge, UK) [20] and Trimmomatic (v0.39, USADELLAB, Aachen, Germany) [21], respectively (Figure 1). To detect potential contamination and perform species identification, we conducted an analysis with Kraken2 (v2.1.3, Johns Hopkins University, Baltimore, MD, USA) [22] using the following parameters: confidence 1, minimum base quality 30, minimum hit groups 2, with the standard plus protozoa and fungi database. Trimmed reads were used to determine genotypic resistance using TB-Profiler (v6.3.0, London School of Hygiene & Tropical Medicine, London, UK) [15] and Mykrobe (v0.13.0, Oxford University, Oxford, UK) [16]. The evolutionary relationships among isolates were reconstructed through implementation of a comprehensive phylogenomic framework. Initial comparative genomic analysis employed the Snippy computational pipeline (v4.6.0, Victorian Bioinformatics Consortium, Melbourne, Australia) [23] for identification of nucleotide polymorphisms and generation of core-genome SNP alignments. For enhanced phylogenetic accuracy, the analytical protocol excluded polymorphic sites within PE/PPE gene families and other repetitive genomic regions with recombination characteristics from the finalized core-genome alignment through application of Gubbins software (v3.3.5, Wellcome Trust Sanger Institute, Cambridge, UK) [24]. Phylogenetic inference was executed via RaxML (v1.2.2, Heidelberg Institute for Theoretical Studies, Heidelberg, Germany) [25], implementing maximum-likelihood methodology under a general time-reversible (GTR) nucleotide substitution model with accommodations for evolutionary rate variability through discrete gamma-distributed rate parameters. Statistical robustness was assessed through 1000 bootstrap replicates. The resultant dendrogram was rendered and graphically enhanced utilizing the Interactive Tree of Life platform (iTOL, v6, University of Hamburg, Hamburg, Germany) [26] to facilitate topological interpretation and evolutionary inference. A minimum spanning tree was generated using Grapetree (v1.5.0, University of Warwick, Coventry, UK) [27], using a 5-SNP cut-off to delineate genomic clusters among the core SNP alignment to identify recent transmission. In silico spoligotyping was conducted using the reads from each isolate with SpoTyping (v2.1, Chinese Academy of Medical Sciences, Beijing, China) [28], and the binary spoligotype code obtained for each isolate was analyzed using the SITVIT2 platform (Pasteur Institute, Paris, France) [29] to identify the family and assign the respective spoligotype international type (SIT).

## 3. Results

### 3.1. Whole-Genome Sequencing (WGS)

The sequenced reads of the selected strains were subjected to quality analysis and filtering. Those sequences with a Phred score below 24 and a length of less than 100 bp were removed. Two of the study strains were excluded from further analysis as they were identified as non-*M. tuberculosis*. One of the studied strains was identified as *M. intracellulare* with 96.05% of reads mapped, and the other was identified as *M. bovis* with 70.4% of reads mapped. The total number of identified SNPs (point mutations differing from H37Rv) ranged between 651 and 2197 (mean: 952). The mean number of small insertions and deletions (indels) detected upon read mapping was 123 and 256 per isolate, respectively.

### 3.2. Phenotypic Susceptibility

The study revealed that out of those isolates tested for first-line anti-TB drug susceptibility, 63.3% (49/77) showed resistance to at least one of the drugs, as shown in Table 1. The remaining 36.3% of the isolates were susceptible to all the tested first-line anti-TB drugs. STR mono resistance was found in 24.5% of the resistant strains, while only 2% of EMB mono resistance was determinate. Eight strains (16.3%) presented resistance to INH and RIF simultaneously and were classified as MDR-TB. The most frequently observed resistance was to INH (*n* = 24) and STR (*n* = 24), followed by PZA (*n* = 16), RIF (*n* = 11), and EMB (*n* = 6).

### 3.3. Genotypic Drug Resistance

The analysis with Mykrobe and TB-Profiler allowed us to determine 39 (50.7%) susceptible strains, 7 (9.1%) MDR strains, and 31 (40.2%) strains resistant to at least one antibiotic (Table S2). The most frequent mutation in the INH-resistant strains was *katG* S135T (61.5%), while in the RIF-resistant strains was *rpoB* S450L (62.5%). For PZA, EMB, and STR, the most frequent mutations were *pncA* L120R, *embB* M306I, and *rpsL* K43R, respectively. According to second-line antibiotic resistance analysis, three strains exhibited genotypic resistance to ofloxacin, moxifloxacin, and levofloxacin due to the presence of the *gyrA* D94G and S91P mutations. Six strains showed resistance to ethionamide caused by mutations in *inhA* and *fabG1*. Two strains exhibited resistance to delamanid due to the *ddn* L49P mutation, and one strain showed resistance to amikacin and kanamycin due to the *rrs* A1401X mutation.

### 3.4. In Silico Typing

**Figure 2:**
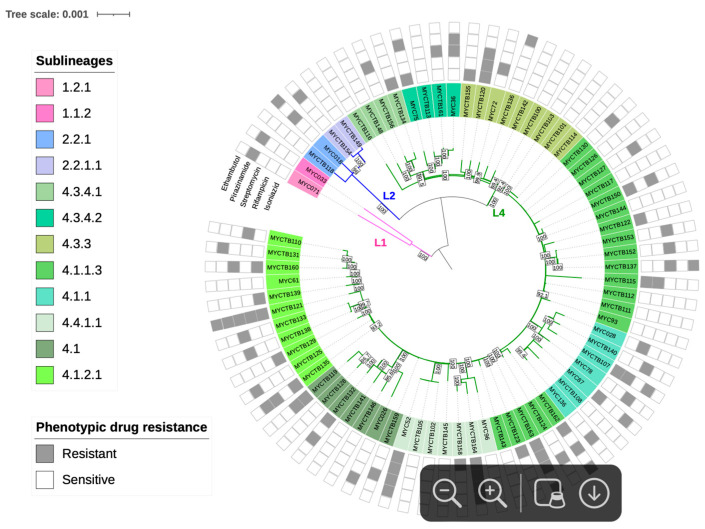
Maximum Likelihood topology of *M. tuberculosis* strains. Branch lengths are proportional to nucleotide substitutions, with the scale bar representing 0.01 substitutions per site. Support values at nodes correspond to bootstrap percentages from 1000 replicates, with values >70% indicated. Colored branches represent different lineages: pink for L1 (Indo-Oceanic), blue for L2 (East-Asian), and green for L4 (Euro-American). Strain identifiers include drug-resistance status (S: susceptible; R: resistant).

Regarding the lineage and sublineage typing based on phylogeny, 2 strains (2.6%) were identified as belonging to lineage 1 (L1), 4 (5.2%) to lineage 2 (L2), and 71 (92.2%) to lineage 4 (L4). These include 2 sublineages of L1 (L1.1.2 and L1.2.1), 2 of L2 (L2.2.1 and L2.2.1.1), and 11 sublineages corresponding to L4 (L4.1, L4.1.1, L4.1.1.3, L4.1.2.1, L4.3.3, L4.3.4.1, L4.3.4.2, and L4.4.1.1) (Figure 2).

The average distance between the 75 MTB strains was 1042 SNPs. To define genomic clusters that might reflect epidemiologically recent transmission, isolates within a maximum distance of 5 SNPs were assigned to genomic clusters (GC). This conservative 5-SNP threshold was selected following the recommendations of Walker et al. (2013) [30] and Meehan et al. (2018) [31], who demonstrated that this threshold has a high specificity for identifying cases of direct or epidemiologically related transmission in tuberculosis studies with whole-genome sequencing. This threshold has also been validated in studies from Latin American settings with similar TB epidemiological patterns to Mexico, including work by Jiménez-Ruano et al. in Veracruz and Acapulco [32].

**Figure 3:**
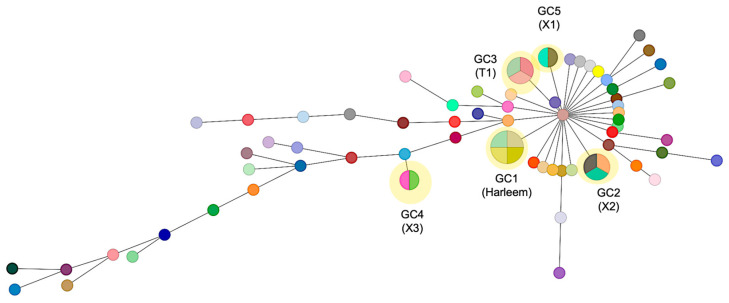
Minimum spanning tree of 75 *M. tuberculosis* strains based on SNP differences. The network displays the genomic relationships between isolates, where each circle (node) represents a strain or group of strains with size proportional to the number of isolates differing by ≤5 SNPs. Lines connecting nodes indicate genetic relationships, with thicker/thinner lines representing closer/more distant relationships based on SNP distances. Genomic clusters (GC1–GC5) are highlighted in yellow, representing likely transmission clusters. GC1 comprises four strains of the Haarlem lineage (SIT47) from Zacatecas; GC2 includes three X2 family strains (two from Zacatecas, one from Michoacán); GC3–GC5 consist of strains from Zacatecas belonging to T1, X3, and X1 families, respectively. Each genomic cluster may contain isolates with different spoligotype patterns, as genomic clustering based on SNPs provides higher resolution than spoligotyping alone.

Using this cutoff criterion, 5 genomic clusters were identified, to which a total of 14 strains (18.7%) were grouped (Figure 3). All GCs were found to belong to independent clades in the phylogeny. GC1 consists of four strains obtained from the state of Zacatecas state and belongs to the Haarlem family; these strains were susceptible to all antibiotics. GC2 comprises two strains isolated from Zacatecas and one from the Michoacán state, classified as MDR and obtained from a patient with digestive TB; by spoligotyping, these strains were assigned to the X2 family. On the other hand, GC3, GC4, and GC5 include strains isolated from Zacatecas and belong to the T1, X3, and X1 families, respectively. The major spoligotype families identified across the whole samples were X1 (22%), T1 (11.7%), and LAM5 (6.5%), while 7.8% had a spoligotype with an undesignated family (Supplementary Materials). Seventy isolates (93.3%) were classified into 22 shared types (SIT numbers), while the remaining five isolates (6.6%) were categorized as not defined. The major SITs determined were SIT 119 (22.6%) and 47 (13.3%). There was a strong concordance between the spoligotype family and the main lineage among the 75 analyzed strains (100%).

## 4. Discussion

Antibiotic resistance in TB is an increasing public health concern in Mexico, reflecting similar challenges faced worldwide. These include delayed diagnosis due to limited access to rapid molecular diagnostics, particularly in rural or resource-poor areas, and restricted availability of second-line drugs and newer treatments such as bedaquiline and delamanid. TB–HIV co-infection complicates treatment and outcomes, and disparities in the quality of care across Mexico exacerbate the issue [2]. Within the Americas, Mexico is the third most affected country in terms of TB cases, behind Brazil and Perú [3]. In this study, we provide robust sublineage classification of MTB strains and identify small transmission groups in a Central Western region of Mexico. Most strains analyzed belong to lineage 4, or the Euro-American lineage (L4) (92.2%), followed by lineage 2, or the East Asian lineage (L2) (5.2%), and lineage 1, or the Indo-Oceanic lineage (L1) (2.6%). Previous studies consistently report L4 as the main driver of TB cases in the region [33,34]. While TB caused by strains belonging to other lineages has been reported, it occurs in much smaller proportions. We identified only 2% of strains belonging to lineage 1, in concordance with previous reports for the Mexican states of Baja California [35], the State of Mexico [36], and Jalisco [37]. L4 is the most globally distributed lineage, prevalent in Europe, the Americas, and regions of Africa and Asia [38]. Genomic studies suggest L4 co-evolution with humans, showcasing adaptations to diverse hosts and environments, along with specific mutations linked to drug resistance, such as in *katG* and *inhA* genes [39]. Although less virulent than L2, the L4 lineage compensates with widespread prevalence and flexibility in immune evasion [40]. Known for high transmissibility, L4 encompasses diverse sublineages, some linked to multidrug-resistant TB (MDR-TB) [41]. These sublineages vary in geographical prevalence, clinical impact, virulence, and transmissibility [39].

The most prevalent sublineage identified in our study was L4.1.1.3 (X-type), which is endemic to Mexico and strongly associated with drug resistance, and it has been proposed to exhibit higher transmissibility compared to other sublineages [41]. Among the 19 L4.1.1.3 strains, 63.15% were resistant to at least one antibiotic, with streptomycin (STR) resistance being the most common (75%). This sublineage is associated with SIT119 and linked to STR-related mutations in this study. In Mexico, L4.1.1.3 is significantly associated with multidrug resistance and possesses unique genetic characteristics that contribute to its pathogenicity and ability to develop resistance. This lineage has a specific geographical distribution, with certain variants (such as SIT 3278) appearing to be endemic to Mexico [32,33]. According to these studies, sublineage 4.1.1.3 (X3) was predominant in 18 (27%) of the isolates, including one genomic cluster formed by eleven multidrug-resistant isolates that appears to be restricted to Mexico. A nationwide phylogenomic surveillance study further noted that this X3 sublineage is distinguished from the rest of the sublineages by containing genes related to pathogenicity and virulence, as well as a gene linked to delamanid [41]. The high rate of drug resistance in this lineage makes it an important target for epidemiological surveillance in the country. Sublineage distribution is well known to vary regionally [42]. Our study reports a predominance of L4.1.1.3 (24.6%), followed by L4.1.2.1 (14.3%), differing from other Mexican regions. For instance, in Jalisco state, WGS-based SNP analysis of 32 samples identified L4.8 as the most frequent sublineage (18.75%) [7], while in Veracruz state, both spoligotyping and MIRU-VNTR analysis of 74 samples identified L4.1.2 as the predominant sublineage [43]. This highlights the importance of understanding the molecular epidemiology of MTB across Mexico to better analyze dispersal dynamics, potential transmission, and population mobility among TB patients. Using a conservative 5-SNP threshold to define transmission clusters, we identified five genomic clusters (GCs), of which GC1 included four strains belonging to SIT47. This is a member of the H1 sublineage that has been detected in Mexico, though it is less common than SIT53 and SIT119 [9,43]. For example, in a study from Monterrey state with 180 isolates, SIT47 accounted for 2.22% of cases [44], suggesting its role in sporadic cases rather than significant outbreaks. However, we identified it in a cluster indicative of recent direct transmission involving four patients. The other GCs include strains corresponding to SIT92 and SIT119. SIT92, X3 family, is a prevalent spoligotype linked to both sporadic and widespread TB outbreaks in South Africa. However, it has been rarely reported in Mexico [42]. Comparatively, SIT119 plays a significant role in TB outbreaks globally, particularly in Mexico, where it is one of the most common spoligotypes in the Central region [9]. Regarding drug resistance, 63.6% of the analyzed strains were resistant to at least one antibiotic, and 16.32% were classified as MDR-TB. According to the WHO Global Tuberculosis Report 2023, Mexico reports approximately 2.5% MDR-TB among new TB cases. However, due to a lack of access to patient data, it was not possible to determine whether our isolates originated from new TB cases or previously treated cases, where higher rates (12–17%) are observed [2]. STR resistance was the most common in our samples (*n* = 24), though only 11 displayed mutations in the STR-associated genes (*rrs*, *rpsL*, *gid*). In the RIF-resistant strains, the most frequent mutation observed was *rpoB* S450L (62.5%). This finding does not align with previous reports that have identified the *rpoB* S531L mutation as the most frequent mutation in RIF-resistant strains in Mexico and other countries [45]. Among the 24 INH-resistant strains identified in this study, the most frequent mutation was *katG* S135T, which has also been reported as the most frequent in Mexico [46]. This may be due to the low fitness of this variant, which could make it more transmissible in bacteria [47].

Several discrepancies between phenotypic and genotypic resistance were observed in our study, with only 42.6% concordance. Similar inconsistencies in resistance determination have previously been reported in Mexico [41]. These discrepancies are unlikely to be caused by technical limitations in sequence analysis, as both TB-Profiler and Mykrobe implement robust algorithms that account for sequencing depth and variant frequency [15,16]. Instead, these discrepancies may be attributed to the limitations of the DST methods, including the use of single-concentration tubes, which establish arbitrary cut-off points and may not detect low-level resistance [41,48]. Additionally, during the culturing process, bacterial populations undergo selection where more fit variants tend to dominate, potentially altering the original proportions of resistant and susceptible bacteria present in the patient sample [49]. Heteroresistance is particularly relevant to our study and deserves special consideration. This phenomenon occurs when a patient is simultaneously infected with susceptible and resistant bacterial populations or when resistance-conferring mutations arise in only a subset of bacteria during treatment [50]. Detecting heteroresistance is challenging—phenotypic testing might identify resistance if the resistant subpopulation grows sufficiently during culture, while standard WGS analysis might miss resistance-conferring mutations if they are present in a small percentage of reads [51]. In our analysis, we identified potential heteroresistance in seven isolates where resistance-associated mutations were present in 10–40% of sequencing reads rather than the >90% mark typically expected for fixed mutations [52]. This pattern was particularly evident for streptomycin resistance-associated mutations, which is consistent with previous studies reporting variable levels of heteroresistance among different antibiotics [53,54]. Future studies employing longitudinal sampling and targeted approaches for specific resistance genes would help elucidate these complex resistance mechanisms in the regional context of Mexico.

While our study provides valuable insights into the genetic diversity and drug resistance profiles of MTB in the Central Western region of Mexico, we acknowledge several limitations. Although our sample size (*n* = 77) is adequate to identify predominant sublineages like L4.1.1.3 (X-type) and L4.1.2.1 (LAM), it limits the statistical power for analyzing rare sublineages such as EAI5, EAI2-Manila, and Beijing, which were represented by only 1–2 strains each. This restricted representation of rare sublineages affects the generalizability of our findings regarding their prevalence and associated resistance patterns. However, the consistent predominance of L4 (92.2%) across our samples aligns with previous reports from Mexico [20,21], suggesting that our core findings regarding the most prevalent lineages are representative of the regional TB epidemiology. Future studies with larger sample sizes, particularly from indigenous communities within these states, would strengthen the epidemiological understanding of rare sublineages in this region.

A significant limitation of this study is the lack of demographic and clinical patient data, such as HIV status, treatment history, urban/rural origin, indigenous status, and socioeconomic factors. Such an information gap restricts our ability to contextualize the identified transmission groups and resistance patterns within their epidemiological framework. For instance, the five genomic clusters detected in this study might be associated with specific risk factors or transmission settings that remain unidentified without patient metadata. Similarly, the high frequency of STR resistance (24.5%) observed might correlate with treatment histories or patient populations. Further studies should prioritize the integration of comprehensive patient metadata, within ethical and privacy constraints, to provide a more nuanced understanding of the risk factors associated with MTB transmission and resistance in this region, particularly regarding vulnerable populations such as indigenous communities that are prevalent in the studied area. However, our findings have direct implications for TB control programs in the Central Western region of Mexico. The five genomic clusters identified, particularly GC1 (SIT47) in Zacatecas, suggest active transmission chains that require targeted interventions including the following: active case finding and thorough contact tracing in geographic areas where genomic clusters were identified (especially in Zacatecas for GC1, GC3, GC4, and GC5); implementation of rapid molecular tests to detect resistance, with emphasis on detecting the predominant *katG* S135T mutation for INH and *rpoB* S450L for RIF; establishment of continuous genomic surveillance systems to monitor the spread of sublineages associated with resistance, particularly L4.1.1.3 (X-type), which showed high levels of STR resistance; and strengthening cross-border and inter-state collaboration for tracking shared strains, such as those observed in GC2 between Zacatecas and Michoacán, to better understand transmission dynamics across regional boundaries.

## 5. Conclusions

This study determined the genetic diversity of MTB in an underexplored region of Mexico using a WGS-based strategy, which offers the highest sensitivity for the phylogenetic classification of isolates. This approach enabled the identification of circulating sublineages in the region, which differ markedly from those reported in other areas of the country, filling the gap in genomic data on MTB strains circulating in the Central Western Region of Mexico. It also highlights the need for a greater number of MTB strains to be sequenced in one of the countries with the highest TB case burden in the Americas.

## Figures and Tables

**Table 1:** Antibiotic resistance frequency in the analyzed *Mycobacterium tuberculosis* strains.

Tested Antibiotic	Frequency (%)
Monoresistant	
INH	9 (18.4)
RIF	3 (6.1)
PZA	6 (12.2)
STR	12 (24.5)
EMB	1 (2)
Drug-resistant type	
TB-RR	3 (6.1)
TB-Hr	16 (32.6)
MDR	8 (16.3)

INH: isoniazid; RIF: rifampin; STR: streptomycin; PZA: pirazinamide; EMB: ethambutol; TB-RR: resistant to rifampin; TB-Hr: resistant to isoniazid and sensitive to rifampin; MDR: resistant to isoniazid and rifampin.

## Data Availability

The original data presented in the study are openly available in SRA at bioproject accession PRJNA880281.

## Supplementary Materials

The following supporting information can be downloaded at: https://www.mdpi.com/article/10.3390/pathogens14060548/s1, Table S1: Drug resistance prediction of Mycobacterium tuberculosis strain analyzed in this study with Mykrobe and TB-profiler; Table S2: Identified spoligotypes, families, and SITs across the studied genomes.

## Abbreviations

The following abbreviations are used in this manuscript:

TB	Tuberculosis
MTB	*Mycobacterium tuberculosis*
L1	Lineage 1
L2	Lineage 2
L3	Lineage 3
L4	Lineage 4
INH	Isoniazid
RIF	Rifampin
STR	Streptomycin
PZA	Prirazinamide
EMB	Ethambutol
OFX	Ofloxacin
MFX	Moxifloxacin
DLM	Delamanid
KAN	Kanamycin
AMK	Amikacin
ETA	Ethambutol
CIP	Ciprofloxacin
LVX	Levofloxacin
LNZ	Linezolid
CPM	Capreomycin
ST	Spoligotype International Type
DR	Drug resistance
MDR TB	Multidrug-resistant tuberculosis
XDR TB	Extensively Drug-Resistant Tuberculosis
